# Novel Screen to Assess Bactericidal Activity of Compounds Against Non-replicating *Mycobacterium abscessus*

**DOI:** 10.3389/fmicb.2018.02417

**Published:** 2018-10-10

**Authors:** Bryan J. Berube, Lina Castro, Dara Russell, Yulia Ovechkina, Tanya Parish

**Affiliations:** TB Discovery Research, Infectious Disease Research Institute, Seattle, WA, United States

**Keywords:** *Mycobacterium abscessus*, nutrient starvation, high-throughput screen, drug discovery, non-replicating, niclosamide

## Abstract

*Mycobacterium abscessus* infections are increasing worldwide. Current drug regimens are largely ineffective, yet the current development pipeline for *M. abscessus* is alarmingly sparse. Traditional discovery efforts for *M. abscessus* assess the capability of a new drug to inhibit bacterial growth under nutrient-rich growth conditions, but this does not predict the impact when used in the clinic. The disconnect between *in vitro* and *in vivo* activity is likely due to the genetic and physiological adaptation of the bacteria to the environmental conditions encountered during infection; these include low oxygen tension and nutrient starvation. We sought to fill a gap in the drug discovery pipeline by establishing an assay to identify novel compounds with bactericidal activity against *M. abscessus* under non-replicating conditions. We developed and validated a novel screen using nutrient starvation to generate a non-replicating state. We used alamarBlue^®^ to measure metabolic activity and demonstrated this correlates with bacterial viability under these conditions. We optimized key parameters and demonstrated reproducibility. Using this assay, we determined that niclosamide was bactericidal against non-replicating bacilli, highlighting its potential to be included in *M. abscessus* regimens. In contrast, most other drugs currently used in the clinic for *M. abscessus* infections, were completely inactive, potentially explaining their poor efficacy. Thus, our assay allows for rapid identification of bactericidal compounds in a model using conditions that are more relevant *in vivo*. This screen can be used in a high-throughput way to identify novel agents with properties that promise an increase in efficacy, while also shortening treatment times.

## Introduction

Infections by nontubercular mycobacteria (NTMs), including *Mycobacterium avium* complex and *Mycobacterium abscessus* complex, are a growing public health concern (Nessar et al., 2012). Traditionally considered an opportunistic pathogen, *M. abscessus* predominantly causes pulmonary disease in patients with cystic fibrosis (CF), chronic obstructive pulmonary disease (COPD), or bronchiectasis. However, recent trends suggest *M. abscessus* infection among otherwise healthy individuals is also on the rise with recent studies showing a 3% increase in prevalence per year (Prevots et al., 2010). Additionally, many cases likely go misdiagnosed as *Mycobacterium tuberculosis* infections due to their similarity in disease presentation and colony morphology upon microscopic examination of sputum smears. This not only underestimates the number of *M. abscessus* infections worldwide but also leads to improper antibacterial treatment (Brown-Elliott et al., 2012).

The increase in NTM infections is troubling in part due to NTMs being particularly refractory to antibiotic treatment with *M. abscessus* resistant to all front-line anti-tuberculosis drugs (Brown-Elliott and Philley, 2017). The American Thoracic Society/Infectious Disease Society of America guidelines state that no current drug regimens are proven efficacious against pulmonary *M. abscessus* infection (Griffith et al., 2007). Drug regimens can be tailored to individuals based on laboratory drug susceptibility testing and individual tolerance to drugs, but generally include a macrolide-based antimicrobial in combination with intravenously administered agents (CF Foundation Clinical Care Guidelines; Griffith et al., 2007). Current guidelines from the Center for Disease Control and Prevention list amikacin, cefoxitin, and clarithromycin as the leading drugs for treatment of *M. abscessus* infection (Lee et al., 2015). These regimens are not only ineffective (29–58% cure rate) but also lead to considerable toxic side-effects due to a typical course of treatment lasting 18–24 months (Jeon et al., 2009; Jarand et al., 2011; Koh et al., 2017; Wu et al., 2018). Antibiotic treatment alone also leads to higher relapse rates than highly invasive surgical procedures involving resection of infected tissue (Griffith et al., 1993; Jarand et al., 2011). These highly invasive procedures should be seen as a last resort, but due to the failure of current drug regimens, these procedures are often the only realistic path to a relapse-free cure (Griffith et al., 2007).

Failure of current drug regimens against *M. abscessus* is likely multi-factorial. One factor is intrinsic antibiotic resistance (Nessar et al., 2012). *M. abscessus* has a waxy cell wall composed of peptidoglycan, arabinogalactan, and long-chain mycolic acids, which is relatively impermeable to antibacterial compounds (Jarlier and Nikaido, 1990; Brennan and Nikaido, 1995). *M. abscessus* also possesses efflux pumps capable of exporting drug, as well as enzymes capable of modifying antibiotics or their targets (Nasiri et al., 2017). These factors make it difficult for drugs not only to enter the cell but also to remain effective if they get past the cell wall. In the few small-scale screens for compounds active against *M. abscessus*, screens suffered from low hit rates compared to other mycobacterial species (Chopra et al., 2011; Gupta et al., 2017; Low et al., 2017).

A major factor in the failure of current drug regimens is the diversity of niches and physiological states encountered by the bacterium during infection, which are not adequately captured with current *in vitro* model systems. The disease pathology of *M. abscessus* infection is similar to that of *M. tuberculosis* (Griffith et al., 2007; Koh, 2017). *M. abscessus* is phagocytosed by macrophages in the lung, but prevents phagosomal acidification and can survive for an extended time (Laencina et al., 2018). During this time physiological and transcriptional changes can lead to phenotypic resistance (tolerance) to drugs (Adams et al., 2011; Larsson et al., 2012). As the infection progresses, NTMs are contained in necrotic granulomas and eventually survive extracellularly in caseum, where they likely experience both low oxygen tensions and/or nutrient starvation (Wu et al., 2018). In *M. tuberculosis*, these conditions lead to a non-replicating state controlled by the DosR regulon, which is characterized by low metabolic activity and enhanced resistance to antibiotics (Wayne and Hayes, 1996; Xie et al., 2005; Leistikow et al., 2010; Sarathy et al., 2018). In this state, *M. tuberculosis* maintains a basal level of metabolic activity to ensure bacterial viability, and the electron transport chain (ETC) is required to maintain the proton-motive force and generate ATP (Rao et al., 2008; Cook et al., 2014).

The ability of *M. abscessus* to survive nutrient starvation or low oxygen tensions has not been established. However, *M. abscessus* does contain a DosR regulon (Gerasimova et al., 2011) and is known to survive in biofilms in the lung of CF patients (Qvist et al., 2015). Additionally, other NTMs have been shown to establish latent infections in patients with subsequent reactivation to active disease (Rosenmeier et al., 1991; Tsilimparis et al., 2014). Together with the similarity in disease progression to TB disease, we believe it is highly likely *M. abscessus* can survive in patients in a dormant state. Previous screens against *M. abscessus* specifically addressed drug activity against actively growing, metabolically active bacteria (Chopra et al., 2011; Gupta et al., 2017; Low et al., 2017). Such efforts will miss compounds which are active against non-replicating or metabolically inactive cells.

The current state of the drug pipeline for NTMs was recently reviewed (Wu et al., 2018), highlighting the need for novel screens to address different physiological conditions encountered by NTMs during infection, including nutrient starvation. Our study aimed to fill part of that gap by developing and validating a novel assay to identify compounds with bactericidal activity against non-replicating *M. abscessus* generated by nutrient starvation. This will allow screening of compound libraries for compounds with sterilizing capabilities under a physiological state likely to be relevant to *in vivo* infection. We demonstrate that a number of drugs currently employed to treat *M. abscessus* infection are inactive in this assay, highlighting the need for drug discovery and development campaigns specifically targeted against *M. abscessus*.

## Materials and Methods

### *Mycobacterium abscessus* Culture

*Mycobacterium abscessus* strain # 103 was purchased directly from BEI Resources (NIAID, NIH) and was cultured in Middlebrook 7H9 medium containing 10% v/v OADC (Oleic Acid, Albumin, Dextrose, Catalase; Becton Dickinson) supplement and 0.05% v/v Tween 80 (7H9-OADC-Tw). Cultures were maintained standing at 37°C. Viable bacteria were determined by colony forming units by plating 10-fold serial dilutions onto Middlebrook 7H10 agar plus 10% v/v OADC supplement. For starvation, mid-log cultures were harvested and resuspended in phosphate-buffered saline (PBS) + 0.05% v/v Tyloxapol (PBS-Tyl) at an OD_590_ of 0.4. Cultures were incubated shaking at 100 rpm for 96 h at 37°C before use. Cultures were harvested and resuspended in PBS-Tyl to an OD_590_ of 0.5 and 50 μL was dispensed into 96-well plates using a Multidrop Combi.

### Preparation of Assay Plates

**FIGURE 1:**
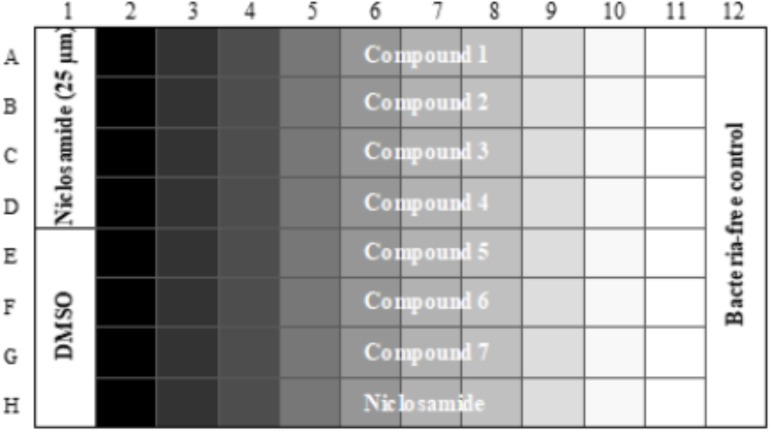
Final assay plate layout. Column 1 contains positive control (Niclosamide) in Rows A–D and negative control (DMSO) in Rows E–H. Column 12 contains bacteria-free control wells. Compounds are tested in Rows A–G as 10-pt serial twofold dilutions. Row H contains a control twofold dilution of niclosamide starting at 25 μM in Column 2.

Compound plates were prepared as described (Ollinger et al., 2013). In brief, 10 mM stock solutions in DMSO were prepared as a 10-point twofold serial dilution in 96-well plates using a Biomek 3000. For the final assay plate, 50 μL of PBS-Tyl was dispensed into each well of a 96-well plate (μCLEAR plates Greiner). Compounds or controls (2 μL of each) were added to each well using a Biomek 4000. The final assay plate contained DMSO as a negative inhibition control and 25 μM niclosamide as a positive control for bacterial killing (**Figure 1**). The final concentration of DMSO in all wells was 2%. Plates were inoculated with 50 μL of bacterial culture; 50 μL of PBS-Tyl was dispensed into column 12 (contamination control). Assay plates were incubated in a humidified incubator for 48 h at 37°C after which 50 μL of 20% v/v alamarBlue^®^ in PBS-Tyl (Bio-Rad) was added using a Multidrop Combi. Plates were incubated at 37°C for 24 h and relative fluorescent units (RFUs) measured at Ex560/Em590 using a BioTek Synergy 4.

### Data Analysis and Statistics

The mean value from the buffer-only wells (background) was subtracted. The coefficient of variance (CV) was calculated as:

$$CV=\frac{\frac{SD}{\sqrt{n}}}{AVG}$$

Z′ was calculated by the following formula:

$$Z^{\prime }=\frac{\left({AVG}_{\max}-\frac{{3SD}_{\max}}{\sqrt{n}})-({AVG}_{\min}-\frac{{3SD}_{\max}}{\sqrt{n}}\right)}{{AVG}_{\max}-{AVG}_{\min}}$$

where AVG is the average RFU, SD is standard deviation, and *n* is the number of controls per plate. The signal-to-background (S/B) ratio is calculated as the mean of the maximum signal wells divided by the mean of the background (no bacteria) wells.

## Results

### Rationale and Establishment of Assay Parameters

We sought to develop a screen to identify compounds with bactericidal activity against non-replicating *M. abscessus*. We used nutrient starvation to generate a non-replicating state, since this is relevant *in vivo* and is technically feasible. We first established the ability of *M. abscessus* to survive under nutrient starvation. We grew *M. abscessus* to mid-log phase and resuspended in buffer to induce complete nutrient starvation. After 96 h, we adjusted the cultures to an OD_590_ of 0.25 (∼2.5 × 10^7^ CFU) and monitored survival. After a further 72 h, bacterial counts were 2.6 ± 0.4 × 10^7^ CFU/mL (*n* = 6, three biological replicates), indicating *M. abscessus* is capable of surviving under non-replicating, nutrient starvation conditions. Our data confirmed that there was no increase in viable bacteria during this time and thus the bacilli are in a non-replicating state.

**FIGURE 2:**
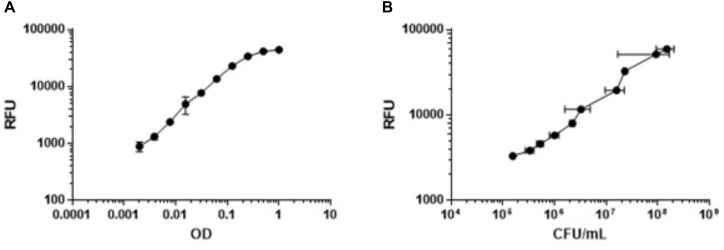
Metabolic activity measured by alamarBlue^®^ correlates with OD_590_ and bacterial viability. Nutrient-starved bacteria were generated in PBS-Tyl. Bacteria were serially diluted twofold and metabolic activity was measured by alamarBlue turnover after 24 h (RFU). **(A)** OD was measured at 590 nm. **(B)** Bacterial viability was measured by determining CFU. Data are average ± SD of three replicates.

We needed a rapid measure of bacterial viability which was amenable to high throughput and required minimal manipulation. Since the ETC is essential under nutrient-starvation, we predicted that respiration would be a good measure of bacterial viability. We tested alamarBlue^®^, which uses the reducing power of the cell to convert resazurin to resorufin, since we could use fluorescence as the readout. We tested the correlation between bacterial viability, measured by optical density, and respiratory output measured by alamarBlue^®^ turnover. We generated nutrient-starved bacteria after incubation in PBS-Tyl and then measured OD_590_ and RFU for twofold serial dilutions, starting at an OD_590_ of 1.0. Optical density correlated in a linear fashion to respiratory output up to an OD_590_ of 0.25 (**Figure 2A** and **Supplementary Figure 1**). Above this level, the RFU signal flattened, likely due to a complete turnover of any alamarBlue^®^ present in the wells or having reached the upper limit of detection of the microplate reader. We confirmed a linear correlation between fluorescence and colony forming units, which determined that a reduction of alamarBlue^®^ signal by 90% is equivalent to ≥1-log reduction in CFU (**Figure 2B** and **Supplementary Figure 2**). We varied a number of parameters (data not shown) and selected the key parameters for the inoculum as 50 μL of a starting culture with a final OD_590_ in the assay plate of 0.25 for assay validation. We established the remaining parameters of the assay as starvation for 96 h, exposure to compounds for 48 h, and incubation with alamarBlue^®^ for 24 h. These parameters gave us sufficient signal to background and signal to noise ratios.

### Reference Compound Activity Under Nutrient–Starvation Conditions

**FIGURE 3:**
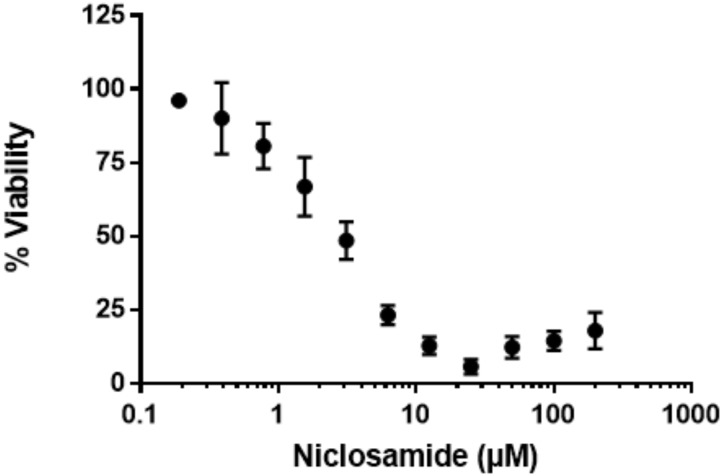
Niclosamide has potent activity against non-replicating bacteria. Nutrient-starved bacteria were exposed to nicolsamide for 72 h. alamarBlue^®^ was added, and RFU measured after 24 h. RFUs were normalized to the DMSO control to express *%* viability. Data are mean ± SD of four independent biological replicates.

We next sought a control compound which would have rapid bactericidal activity against *M. abscessus* under starvation conditions. We selected a number of compounds based on current treatment guidelines for *M. abscessus* infection, as well as compounds known to be active against non-replicating bacteria. Surprisingly, almost all the compounds we tested were inactive, including most of those currently used as part of *M. abscessus* drug regimens (**Table 1**). Only three of the tested compounds inhibited alamarBlue^®^ turnover by at least 90%, i.e., MBC_90_ (**Table 1**). Kanamycin and amikacin had MBC_90_ values of 39 and 16 μM, respectively, while niclosamide was the most effective compound with a MBC_90_ of 8.4 μM (**Table 1**) and maximal activity at 25 μM (**Figure 3**).

**Table 1:** Activity of drugs against non-replicating *M. abscessus*.

Compound	MBC_90_ (μM)
Niclosamide	8.4 ± 0.5
Amikacin	16 ± 0.5
Kanamycin	39 ± 2.3
Ebselen	>100
Cefoxitin	>200
Tiacumicin	>200
Rifampicin	>200
Clarithromycin	>200
Imipenem	>200
Linezolid	>200
Azithromycin	>200
Ethambutol	>200
Ciprofloxacin	>200
Levofloxacin	>200
Moxifloxacin	>200


*Nutrient-starved bacteria were exposed to compounds for 48 h, alamarBlue was added, and RFU measured after 24 h. MBC_*90*_ is the concentration required to reduce alamarBlue turnover by 90% as determined by the Gompertz equation. Data are mean ± SD of at least two independent biological replicates.*

### Confirmation of Assay Serving as a Measure of Bactericidal Activity

**FIGURE 4:**
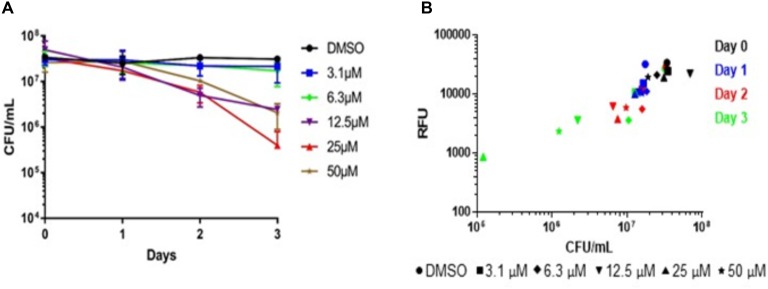
Metabolic activity correlates with bactericidal activity. Nutrient-starved bacteria were exposed to compounds for 24–72 h, alamarBlue^®^ was added and RFU measured after 24 h. Aliquots from the same samples were used to determine: **(A)** Bacterial viability (CFU; data are the average ± SD of 2 biological replicates); **(B)** metabolic activity.

To confirm our screen’s ability to serve as a measure of bacterial killing, we assayed *M. abscessus* survival over time in the presence of niclosamide. Concentrations of niclosamide below the MBC_90_ (3.1and 6.3 μM) had minimal activity (**Figure 4A**), while concentrations above the MBC_90_ had >1-log reduction in CFU within three days; 25 μM reduced bacterial viability by 1.5-log CFU (**Figure 4A**). This data corresponds to the concentration–response curve seen in **Figure 3**, where 25 μM niclosamide caused the greatest reduction in alamarBlue^®^ turnover. At higher concentrations (50 and 100 μM), niclosamide had reduced activity due to solubility issues. We measured alamarBlue^®^ turnover each day. **Figure 4B** shows bacterial viability correlates in a linear fashion with RFU in our assay (*R*^2^ = 0.59). Over time, niclosamide killed *M. abscessus*, and there was a concomitant decrease in alamarBlue^®^ turnover (**Figure 4B**). These data confirm that alamarBlue^®^ can be used as a readout for bacterial viability.

### Validation of High-Throughput Screen

**FIGURE 5:**
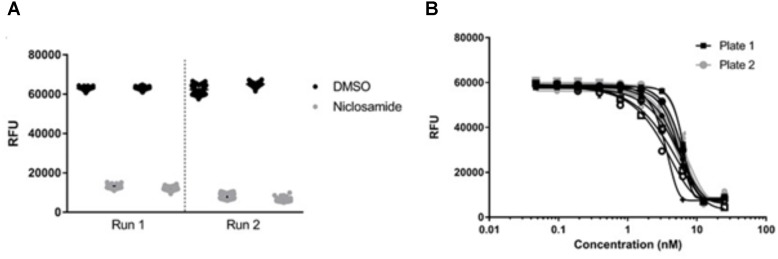
Validation of high-throughput screen. **(A)** Metabolic activity measured by alamarBlue^®^ turnover was plotted for individual plates from each of two separate biological runs. **(B)** Activity of niclosamide was determined in 16 technical replicates across two different plates. Line of best fit was plotted by the Gompertz equation. Data are representative of three independent biological replicates.

We tested the reproducibility of our screen by testing for intra-experiment and inter-experiment variability (Iversen et al., 2004). We ran three separate experiments each containing duplicate plates with full 96-well plates of the maximum signal (2% DMSO), minimum signal (25 μM niclosamide) and dose–response curves (niclosamide 10-point, twofold dilutions). Inter- and intra-assay variability were low (**Figure 5A**). The average CV across all DMSO control plates was 11%, while average CV for niclosamide plates was only 5% (**Table 2**), both well below the minimum standard for CV of 20% specified in the assay guidance manual (Iversen et al., 2004). The curves for niclosamide were consistent within and across runs (**Figure 5B**) with an average MBC_90_ of 9.1 ± 3.2 μM and MBC_50_ of 5.0 ± 0.9 μM across 48 replicates and an average Z′ score for these plates of 0.84 ± 0.16 (*n* = 6; **Table 2**). Thus, the assay is reproducible and suitable for high throughput screening.

**Table 2:** Assay reproducibility and robustness testing.

Run	Plate	Z′	% CV DMSO	% CV Niclosamide	S/B
1	1	0.82	1	6	23
	2	0.94	1	7	23
2	1	0.90	3	16	22
	2	0.90	2	7	23
3	1	0.50	13	15	18
	2	0.96	11	15	18
Mean		0.84	5	11	21
SD		0.16	5	4	2


*The final assay was run in three independent runs containing two plates each of minimum signal (2% DMSO), maximum signal (25 μM niclosamide), and niclosamide CRCs with positive and negative controls. Nutrient-starved bacteria were exposed to compounds for 48 h, alamarBlue^®^ was added, and RFU measured after 24 h. Z′, % CV, and signal/background (S/B) ratio were calculated for each experimental run.*

## Discussion

The drug development pipeline for *M. abscessus* is extremely sparse at a time when infections worldwide are on the rise and current drug regimens are highly ineffective (Griffith et al., 2007). Recently, a call was issued for the development of novel assays to assess the efficacy of compounds against *M. abscessus* under various physiological states with the hope these assays might lead to hits that are more physiologically relevant, and thus more likely to be successful in the clinic. In this work, we describe one such assay that specifically identifies compounds that are bactericidal to nutrient-starved *M. abscessus*.

Nearly all tested compounds were completely inactive against non-replicating *M. abscessus* (**Table 1**). This could be due to an inability of the compounds to penetrate the *M. abscessus* cell wall or to their inactivity under nutrient-starvation conditions. Surprisingly, we found only one of the drugs recommended by the Center for Disease Control and Prevention to treat *M. abscessus* was active in this assay. Amikacin, an aminoglycoside inhibitor of the 30S ribosomal subunit, had a MBC_90_ of 16 μM, indicating this drug actively targets non-replicating *M. abscessus*. On the other hand, cefoxitin and clarithromycin were completely inactive (**Table 1**). Inactivity of cefoxitin is potentially explained by its mechanism of action. As a beta-lactam antibiotic, cefoxitin targets the cell wall of actively dividing cells, a potential liability against non-replicating *M. abscessus*. Clarithromycin was also inactive in this assay, despite being a protein synthesis inhibitor like amikacin. One possible explanation is the known induction of clarithromycin resistance in some *M. abscessus* isolates (Benwill and Wallace, 2014; Koh, 2017). Whatever the mechanism, it is troubling two of the three recommended treatments for *M. abscessus* are inactive. It is tempting to speculate the general inactivity of the tested compounds against non-replicating *M. abscessus* may play a role in the failure of these drugs to adequately treat patients. More work needs to be done to assess their efficacy under other physiological states likely encountered by *M. abscessus* during infection.

We did identify two other compounds capable of killing non-replicating *M. abscessus*, namely kanamycin and niclosamide. Kanamycin, like amikacin, is an aminoglycoside inhibitor of ribosomal translocation (Misumi and Tanaka, 1980) and is commonly used as a second-line drug in regimens to treat multidrug-resistant tuberculosis (MDR-TB). However, kanamycin has considerable negative side effects shared with amikacin (Lane et al., 1977; Jiang et al., 2017). First, kanamycin is not orally bioavailable; it is given as an injectable drug, making its delivery more difficult than drugs available as pills. Second, patients undergoing prolonged aminoglycoside treatment can experience nephrotoxicity, as well as significant hearing loss, and thus must undergo repeated testing to determine whether treatment can continue (Ariano et al., 2008; Al-Malky et al., 2015; Garinis et al., 2017). With current drug regimens for *M. abscessus* lasting 18–24 months, kanamycin is not an ideal drug to be included in a new regimen.

Niclosamide was the most effective compound in this assay, causing 1–2 log-CFU kill in just three days (**Figure 4**). Niclosamide, an anti-tapeworm drug, is an inhibitor of oxidative phosphorylation, potentially working by inhibiting production of adenosine triphosphate (ATP) (Frayha et al., 1997; Chen et al., 2018). Niclosamide is an essential medicine according to the World Health Organization and is available as a chewable tablet (World Health Organization [WHO], 2017), although there are issues with solubility, absorption, and distribution to tissues (Chen et al., 2018). Recent work has been done to improve pharmacokinetic (PK) and pharmacodynamic (PD) properties of niclosamide, which could lead to its use to treat various bacterial infections (Mook et al., 2015). Our work suggests niclosamide could be used as part of a drug regimen to treat *M. abscessus*. If PK/PD properties were suitable to provide adequate exposure in the lungs, niclosamide might be able to target non-replicating bacilli residing in granulomas or caseum and help clear the infection.

## Conclusion

In conclusion, we have developed a new assay to find anti-mycobacterial agents with activity against non-replicating bacilli, where positive hits can be followed up for bactericidal activity by plating for CFU. This assay allows us to screen for compounds that are bactericidal within a short period (3 days), increasing the possibility that we can identify rapidly bactericidal agents that could have an impact on the treatment of *M. abscessus* infections. We used this assay to determine that niclosamide is a rapidly bactericidal agent against non-replicating *M. abscessus*.

## Author Contributions

BB and TP conceived the experiments. BB, LC, YO, and TP designed all the experiments. BB, LC, DR performed the experiments. BB, LC, and TP wrote the manuscript. All authors edited and approved the final manuscript.

## Conflict of Interest Statement

The authors declare that the research was conducted in the absence of any commercial or financial relationships that could be construed as a potential conflict of interest.
