# Supplementary material for: Novel Screen to Assess Bactericidal Activity of Compounds Against Non-replicating Mycobacterium abscessus
Source: Front Microbiol. 2018 Oct 10;9:2417. doi: 10.3389/fmicb.2018.02417 (PMC6191478; doi:10.3389/fmicb.2018.02417)
Supplement: Supplementary file 1 [file Data_Sheet_1.PDF]

Supplemental Figure 1

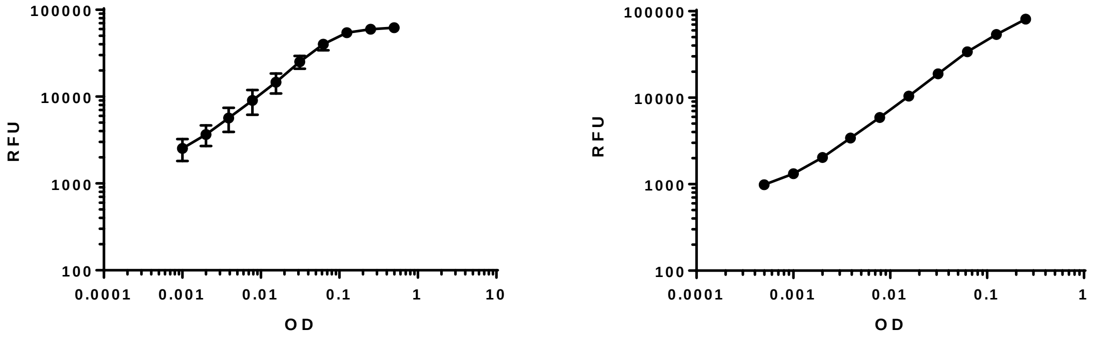

Replicates of Figure 2A. alamarBlue turnover was measured in nutrient-starved *M. abscessus* in 2-fold dilutions beginning at an OD_{590} of 0.5 (left) or 0.25 (right) to test for linearity of the assay.

Supplemental Figure 2

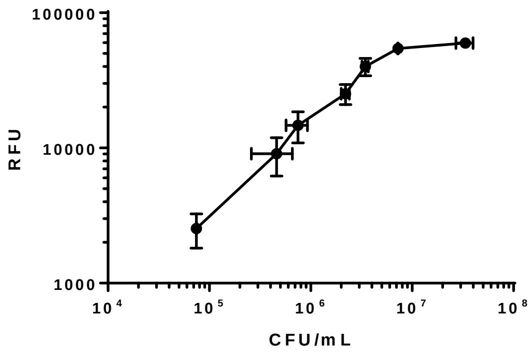

Replicate of Figure 2B. Nutrient-starved *M. abscessus* was serially 2-fold beginning at an OD_{590} of 0.25. Viable biacteria as measured by CFUs was correlated to alamarBlue turnover by RFUs.
